# *Faecalibacterium prausnitzii* Colonization Attenuates Gut Inflammation and Epithelial Damage in a DSS-Induced Colitis Mice Model

**DOI:** 10.1155/mi/7280675

**Published:** 2025-03-10

**Authors:** Meng-Chuan Liu, Yu-An Shu, Yu-Chin Wang, Hsiu-Ying Tseng, Meng-Jia Li, Yu-Ting Yu, Hsiu-Chi Cheng, Pei-Jane Tsai, Yao-Jong Yang

**Affiliations:** ^1^Department of Pediatrics, National Cheng Kung University Hospital, College of Medicine, National Cheng Kung University, Tainan, Taiwan; ^2^Department of Medical Laboratory Science and Biotechnology and Center of Infectious Disease and Signaling Research, National Cheng Kung University, Tainan, Taiwan; ^3^Institute of Basic Medical Sciences, College of Medicine, National Cheng Kung University, Tainan, Taiwan; ^4^Department of Pathology, Chung Shan Medical University Hospital, School of Medicine, Chung Shan Medical University, Taichung, Taiwan; ^5^Department of Internal Medicine, National Cheng Kung University Hospital, College of Medicine, National Cheng Kung University, Tainan, Taiwan; ^6^Institute of Clinical Medicine, National Cheng Kung University Hospital, College of Medicine, National Cheng Kung University, Tainan, Taiwan

**Keywords:** animal model, colitis, colonization, DSS, *Faecalibacterium prausnitzii*

## Abstract

**Background:** Reduction of *Faecalibacterium prausnitzii* abundance is related to inflammatory bowel diseases (IBDs), and supplement of it exists protective effects.

**Aim:** This study aimed to establish a *F. prausnitzii*-colonized mouse model and investigate that the presence of *F. prausnitzii* in the gut can ameliorate the severity of dextran sulfate sodium (DSS)-induced colitis.

**Methods:** A *F. prausnitzii* (ATCC 27768) strain was maintained on the PS-BHI agar plates and manipulated in a strictly anaerobic chamber. A *F. prausnitzii*-colonized C57BL/6 mice model was tested by a rectal enema with 1 × 10^9^ bacteria/day for 3 days. The 5% DSS was added to drinking water for 3 days to induce colitis and diarrhea in experimental mice. The clinical, cytological, and histological severities were compared between groups.

**Results:** The *F. prausnitzii*-colonized mice model was successfully established via rectal enema with the property of transfer to offspring. DSS treatment altered gut microbiota and significantly attenuated the abundance of *F. prausnitzii* in colonized mice. Mice with *F. prausnitzii* colonization had significantly improved weight loss, anal bleeding, stool consistency, cecum weight, colon length, and serum amyloid A (SAA) level than those without after DSS treatment. Furthermore, the *F. prausnitzii*-colonized mice significantly reduced the transcription levels of TNF-α, INF-γ and IL-18, and epithelial damage and PMN infiltration in the lamina propria and had better preservation of goblet cells than the control group.

**Conclusion:** We have successfully established a mouse model colonized with *F. prausnitzii* via rectal enema administration and showed colonization of *F. prausnitzii* in the gut has a protective effect against DSS-induced colitis.

## 1. Introduction

Inflammatory bowel disease (IBD) is a spectrum of disorders characterized by chronic or relapsing inflammation of the gastrointestinal tract. Crohn's disease (CD), ulcerative colitis (UC), and IBD unclassified (IBDU) represented the three main subtypes of IBD leading significant morbidity [1]. Both incidence and prevalence of IBD are increasing worldwide [2]. What is worse is that IBD is life-long and is not curable with current medical knowledge. The disease is a condition driven by the complex interaction between genetic susceptibility, gut microbiota dysbiosis, gut barrier dysfunction, environmental factors, and host immune system [3]. Novel treatments are developed to target the complex mechanism behind the pathogenesis of IBD.

Studies have shown that dysbiosis of the gut microbiota is correlated with the development and severity of IBDs [4]. Nishida et al. [5] reported that a decrease of Firmicutes and an increase of Proteobacteria plays a role in the pathogenesis of IBD. *Faecalibacterium prausnitzii* belonging to the phylum of Firmicutes is a dominant colonic commensal bacterium, accounting for 5% of the total gut microbiota in healthy adults [6]. This microorganism is a rod-shaped, nonmotile, nonspore forming, and strictly anaerobic bacterium. Several clinical observations found that reduction of the abundance of *F. prausnitzii* is related to patients with IBD, including UC and CD [7–9]. Furthermore, animal studies have shown that administration of *F. prausnitzii* or its supernatant exhibited protective effects on intestinal permeability and cytokines production in chemical-induced mice models [10, 11]. These results indicate that the *F. prausnitzii* may express the anti-inflammatory effect for gut inflammatory disease and may play a critical role in the pathogenesis of IBD.

By the direct toxic effect on epithelial cells, the dextran sulfate sodium (DSS) has shown to induce the injury of epithelial integrity and increase the proinflammatory cytokines in mice models [11]. DSS-induced colitis mice model with features including epithelial ulceration, submucosal edema, and inflammatory cell infiltration is considered to represent human IBDs. To investigate the effect of colonization of *F. prausnitzii* on IBD, we created a novel *F. prausnitzii*-colonized mice model and validated the protective effects for DSS-induced colitis.

## 2. Methods

### 2.1. Bacteria and Culture Condition

A *F. prausnitzii* (ATCC 27768) strain was purchased from the Bioresource Collection and Research Center (BCRC), Taiwan. The bacterium was grown and manipulated in a strictly anaerobic chamber (90% N_2_, 5% CO_2_, and 5% H_2_) and was maintained on the YPS-BHI (brain–heart infusion medium supplemented with 0.5% yeast extract, 0.1% potato starch [Difco]) agar plates and suspended in the broth before tests. The YPS-BHI medium (per 1000 mL) consists of brain heart infusion extract (37 g) (BD Difco), yeast extract (3 g) (Condalab), potato starch (1 g) (Sigma), glucose (3 g) (Sigma), L-cysteine HCl (0.5 g) (Bio-basic), sodium acetate (3 g) (Sigma), and resazurin (0.025%, 4 mL) (Sigma).

### 2.2. *F. prausnitzii*-Colonized Mice Model

**Figure 1:**
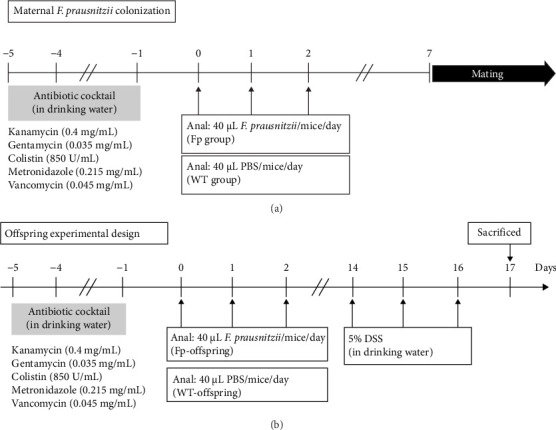
The diagram of protocol for establishing *F. prausnitzii* colonization in maternal and their offspring C57BL/6 mice. (A) Antibiotic cocktail was given to mice in drinking water from day −5 to day −1. The female mice were administrated with 40 μL phosphate-buffered saline (PBS) (WT group) or *F. prausnitzii* (1 × 10^9^ bacteria) suspension (Fp group) through anal injection once a day from day 0 to day 2. The fresh stool samples were collected to verify successful *F. prausnitzii* colonization or not by PCR. (B) Once successful colonization was verified in female mice, wild-type males were caged with females. After mating, neonatal mice birth, the maternal mice were housed with their offspring at room temperature until weaning (3 weeks). From day 14 to day 16, 5% dextran sulfate sodium (DSS) was added in drinking water to induce colitis. All mice were sacrificed at day 17 for collection of serum, colonic tissue, and feces samples. *Note:* WT-offspring, offspring from the WT group mother; Fp-offspring, offspring from the Fp group mother.

To establish the *F. prausnitzii* colonization mice model, *F. prausnitzii* ATCC 27768 was cultured from −80°C stock on YPS-BHI agar for 48 h at 37°C in an anaerobic conditions. Single colony was transferred to 100 mL YPS-BHI broth and cultured for 48 h for use. The colonization protocol was modified from that of our previous mice model [12]. In brief, the wild-type (WT) female C57BL/6J mice (*n* = 4 in each group) were obtained from the National Laboratory Animal Center. All our animal studies were performed following a protocol approved by the Institutional Animal Care and Use Committee (IACUC) of the National Cheng Kung University (NCKU). The experimental mice study was approval by the IACUC of NCKU (approval NCKU-IACUC-109294) and the Biosafety and Radiation Safety Management Division of NCKU. Antibiotic cocktail, containing 0.045 mg/mL vancomycin, 0.215 mg/mL metronidazole, 0.4 mg/mL kanamycin, 0.035 mg/mL gentamicin, and 0.057 mg/mL colistin, was gave to mice in drinking water from day −5 to day −1 (Figure 1A). The female mice were randomly separated into two groups administrated with phosphate-buffered saline (PBS, WT group) or *F. prausnitzii* at day 0 (Fp group). A 40 μL *F. prausnitzii* suspension (1 × 10^9^ bacteria) or 40 μL PBS was given through anal injection once a day from day 0 to day 2. On day 7, fresh stool samples were collected to verify successful *F. prausnitzii* colonization or not by PCR using primers as follows: forward, 5′- AACTTYATYTCCATCAACAAYGC-3′, and reverse, 5′- CAGATRAAGCTCTTGCCGC-3′. Once successful colonization was verified, WT males were caged with females. Mating was determined by a vaginal plug and marked as embryonic day. After birth, the maternal mice were housed with their offspring at room temperature until weaning. Offspring from the WT group mother were referred to as WT-offspring, and those from the Fp group mother were referred as Fp-offspring.

### 2.3. Experimental Design

Figure 1B showed the experimental *F. prausnitzii* colonization offspring ameliorated DSS-induced colitis than noncolonized controls. Fresh stool specimens were collected from offspring at postnatal 8 weeks. All offspring were given with antibiotic cocktail at week 8 after birth from day −5 to day −1. From day 0 to day 2, *F. prausnitzii* suspension (40 μL containing 1 × 10 [9] bacteria/day) was inoculated to Fp-offspring through anal injection once a day to strength the amount of *F. prausnitzii* in colon; PBS (40 μL/day) was given to WT-offspring also through anal injection. To investigate the protective effect of *F. prausnitzii* colonization in DSS-induced colitis mice, the 5% DSS was added in the drinking water since day 14 to day 16 for inducing colitis. The offspring were randomly assigned to one of the four groups (*n* = 6 in each group): The control group was WT-offspring, and no DSS was added in drinking water. The DSS group was WT-offspring given with DSS in drinking water. The Fp group was Fp-offspring, and no DSS was added in drinking water. The Fp + DSS group was Fp-offspring given with drinking water containing DSS.

The body weight, stool consistency, and anal bleeding of offspring were recorded every day. The score of stool consistency was defined as score 0, well-formed pellets; score 1, semiformed stools that did not adhere to the anus; score 2, semiformed stools that adhered to the anus; and score 3, liquid stools [12]. Fresh stool was collected again on day 17. All mice were sacrificed on day 17, and the blood sample was collected from the heart puncture for biochemistry examination; colon length and cecum weight were measured. After separation of the total colon, half of them were fixed with formalin for histopathologic examination, while the other half was grinded to prepare RNA for measuring tumor necrosis factor-α (TNF-α), interleukin (IL)-1β, macrophage inflammatory protein-2 (MIP-2), interferon (IFN)-γ, IL-17, and IL-18 levels using primers in Table S1.

### 2.4. Sequencing for Fecal Microbiota

We conducted quantitative PCR analysis on the fecal samples of WT-offspring and Fp-offspring mice, as well as Fp-offspring post-DSS treatment, utilizing taxon-specific primer sets tailored for the following phyla: Bacteroidota, Bacillota, Actinomycetota, Pseudomonadota, and Verrucomicrobiota (Table S1). The qPCR was carried out on these samples using an Applied Biosystems StepOne instrument (Waltham, MA) with SYBR Green assay for quantification of all samples following modification of a previously study [13]. The raw results were normalized by the signal of Eubacteria and calculated using the delta–delta–comparative threshold (Ct) method [14].

### 2.5. Histopathologic Examination

For histological examination, the colon tissue was washed and open, fixed in 10% formaldehyde solution, embedded in paraffin, cut into sections (4 µm), and stained with hematoxylin and eosin (H&E) and Periodic acid–Schiff (PAS). The colons from the sacrificed mice were opened longitudinally along the mesenteric axis, and one-half was fixed in buffered formalin. The fixed tissue was embed in paraffin and 3-μm-thick sections were prepared for H&E stain. An experienced pathologist blinded assessed the stained sections using a scale designed from Farooq and Stadnyk [15]. The pathology included the presence (or not) of submucosal edema, the extent of mucosal ulceration, crypt loss, and PMN infiltration.

### 2.6. Enzyme-Linked Immunosorbent Assay (ELISA)

The serum amyloid A (SAA) of serum in mice was quantified using Serum Amyloid A ELISA kits (“PHASE”, Tridelta Development Ltd. Ireland). The procedure was guided by manufactory instruction.

### 2.7. Quantitative Real-Time Reverse Transcription PCR

Total RNA was extracted from colonic tissue, and 1 μg of total RNA was reversely transcribed using the Invitrogen M-MLV Reverse Transcriptase with random decamers as primer. To melt the RNA secondary structure, we incubated the RNA, random decamer (0.5 μg, 1 μL), 2.5 mM dNTP (4 μL), and distilled water (to 12 μL) at 65°C for 5 min, cooled to 42°C, then added 5X First-Strand Buffer (4 μL), 0.1M DTT (2 μL), distilled water (1 μL), and incubated at 37°C for 2 min. Then we added M-MLV Reverse Transcriptase (200 units, 1 μL) and left it at 37°C for 50 min. Next, we heated the mixture to 70°C for 15 min to inactivate the reaction. Real-time PCR amplification was performed using Power SYBR qRT-PCR kits (Applied Biosystems, Foster City, CA) on QuantStudio 5 analyzer (Applied Biosystems) for primers: TNF-α, IL-1β, IL-17, IL-18, IFN-γ, and MIP-2. Relative amount of target messenger RNA (mRNA) was determined using the Ct method by normalizing.

### 2.8. Statistical Analysis

Results are expressed as mean ± standard deviation (SD). In comparison of the statistically significant difference between multiple groups, the one-way ANOVA with a post hoc test was used. Differences between two samples were checked for statistical significance using the independent *t* test or Mann–Whitney *U* test. A *p* value < 0.05 was defined as statistical significance.

## 3. Results

### 3.1. Establishment of *F. prausnitzii* Colonization in Mice Model

**Figure 2:**
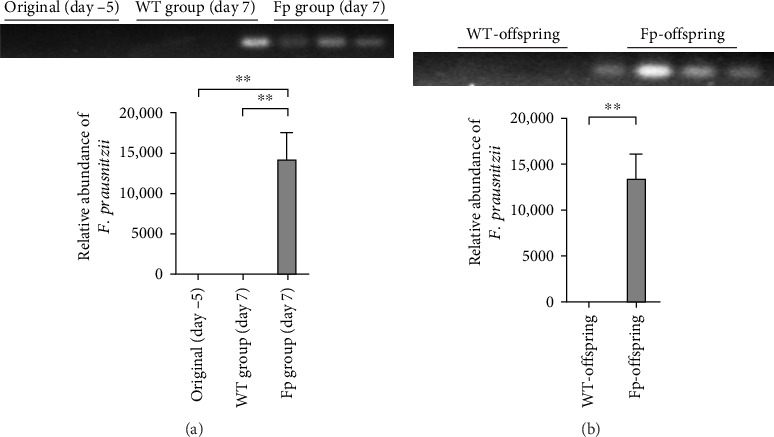
The relative abundance of *F. prausnitzii* was detected by PCR from feces. (A) on the 7^th^ day postinoculation, a positive *F. prausnitzii* DNA was found in Fp group. (B) Offspring mice nourished and housed with *F. prausnitzii*-colonized mother showed colonization of *F. prausnitzii* in the colon at postnatal week 8 (*⁣*^*∗*^*p* < 0.05; *⁣*^*∗∗*^*p* < 0.01).

Because *F. prausnitzii* was susceptible to pH value and bile salt, oral gavage was failed to colonize this bacterium to experimental mice (data not shown). Therefore, we inoculated *F. prausnitzii* suspension via rectal enema as protocol.  Figure 2 showed the abundance of *F. prausnitzii* from feces in maternal and offspring mice. Prior to the first administration (day –5) and postinoculation day 7, *F. prausnitzii* was not detected in the feces of the WT C57BL/6 mice (WT group). The successful colonization of *F. prausnitzii* in the colon was found at the postinoculation day 7 (Figure 2A) in the Fp group. Furthermore, to evaluate whether it exists the mother-to-infant transfer of *F. prausnitzii*, the DNA of *F. prausnitzii* was detected in the Fp-offsrping at postnatal week 8 and was absent in the WT-offspring (Figure 2B).

### 3.2. *F. prausnitzii* Colonization Improves Body Weight Loss, Stool Consistency, and Rectal Bleeding After DSS Treatment

**Figure 3:**
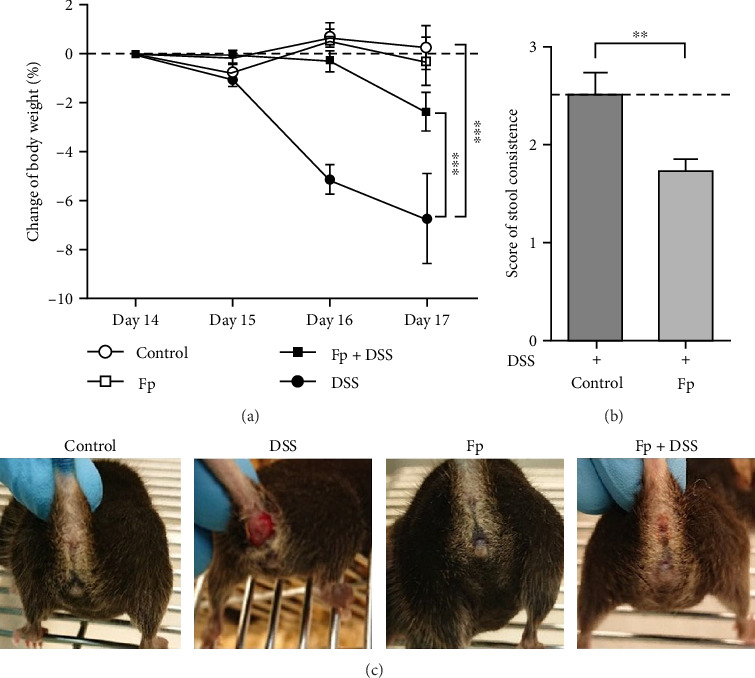
*F. prausnitzii* colonization improves anal bleeding after dextran sulfate sodium (DSS) treatment in offspring mice with and without *F. prausnitzii* colonization. (A) The body weight of the mice was checked every day and expressed as the percentage change relative to the initial weight on DSS administration (day 14). (B) The mean score of stool consistence was compared between mice with and without *F. prausnitzii* colonization at day 17. (C) Rectal bleeding observed between four groups. The error bars represent standard errors (*⁣*^*∗∗*^*p*  < 0.01; *⁣*^*∗∗∗*^*p*  < 0.001).

In comparison of the protective effect of *F. prausnitzii* colonization in DSS colitis mice, daily body weight change (BWC) (daily weight–weight of day 14), stool consistency score (day 17), and rectal bleeding were compared between Fp-offspring (Fp) and WT-offspring (control) with and without DSS (Figure 3). Figure 3A showed the percentage of BWCs from day 14 (DSS day 1) to day 17 (end of study) in the four groups. At day 17, WT-offspring mice with DSS treatment had a significant reduction of BWC (%) than controls (*p* < 0.001). However, Fp-offspring mice significantly improved the BWC as compared to WT-offspring mice with DSS treatment (*p* < 0.001). The mean stool consistence was shown in Figure 3B between WT-offspring and Fp-offspring mice after DSS treatment. The result showed mice with *F. prausnitzii* colonization had significantly less score than those without colonization (*p* < 0.01). In observation of rectal bleeding in mice, obvious fresh blood from anus was found in mice of WT-offspring and less severe in those Fp-offspring after DSS treatment (Figure 3C).

### 3.3. *F. prausnitzii* Colonization Preserves the Cecum Weight and Colon Length and Reduces Systemic Inflammation in DSS-Induced Colitis Mice

**Figure 4:**
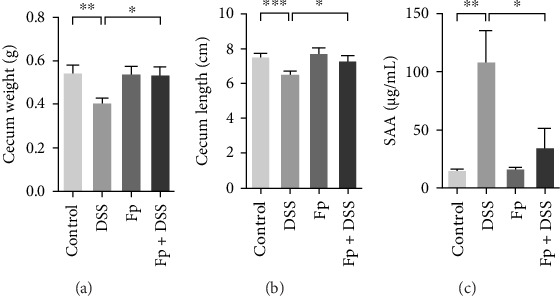
*F. prausnitzii* colonization improved cecum weight (A) and colon length (B) and reduced the serum amyloid A (SAA) level (C) in dextran sulfate sodium (DSS) colitis mice. The error bars represent standard errors (*⁣*^*∗*^*p* < 0.05; *⁣*^*∗∗*^*p* < 0.01; *⁣*^*∗∗∗*^*p* < 0.001).

The cecum weight, colon length, and SAA level, as a marker of systemic inflammation, were measured (Figure 4). On day 17, the appearance of the cecum and colon from the sacrificed mice was observed. DSS treatment significantly reduced the cecum weight and the colon length than controls (Figure 4A,B). Furthermore, *F. prausnitzii*-colonized mice significantly improved cecum weight (*p* < 0.05) and colon length (*p* < 0.05) than those noncolonized mice after DSS treatment. Figure 4C showed the SAA level significantly elevated after DSS treatment in noncolonized (DSS) mice (*p* < 0.05); nevertheless, *F. prausnitzii* colonization (Fp + DSS) significantly reduced the SAA level than noncolonized mice (*p* < 0.05).

### 3.4. *F. prausnitzii* Colonization Attenuates Histopathological Severity in DSS-Induced Colitis Mice

**Figure 5:**
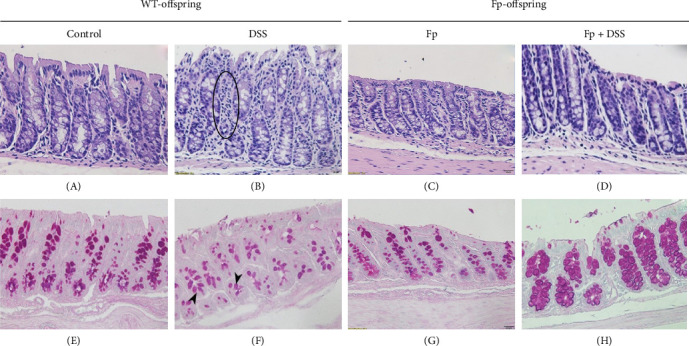
Histopathological features in the mice colon tissue, represented by hematoxylin and eosin (H&E) stained of the resected colons in the control group (A), dextran sulfate sodium (DSS) group (B), Fp group (C), and Fp + DSS group (D). Periodic acid–Schiff (PAS) stain of the colonic tissue in the control group (E), DSS group (F), Fp group (G), and Fp + DSS group (H). Magnifications: ×200. In DSS colitis mice, there was mucosal epithelial damage, inflammatory expansion of the lamina propria (B, circle), and reduced goblet cell numbers (F, arrow heads). DSS mice with *F. prausnitzii* colonization were characterized by minimal epithelial damage and immune cell infiltration (D) and nearly normal goblet cell amount (H).

Figure 5 showed the histological manifestations between *F. prausnitzii*-colonized and noncolonized mice after DSS treatment. The colonic tissues of the control group (Figure 5A,E) as well as *F. prausnitzii*-colonized group (Figure 5C,G) displayed intact mucosa and submucosa without inflammatory cells and normal goblet cell amount. WT-offspring mice with colitis evoked by DSS showed severe mucosal epithelial damage and crypt withering, and the lamina propria was expanded by neutrophils and lymphocytes (Figure 5B, circle). PAS staining revealed the reduction of numbers and size of goblet cells (Figure 5F, arrow heads). In contrast, among *F. prausnitzii*-colonized mice treated with DSS (Fp-DSS), the colonic tissues were characterized by minimal epithelial damage and less immune cell infiltration (Figure 5D). Moreover, PAS staining revealed the preservation of goblet cells (Figure 5H). These results disclosed that *F. prausnitzii* colonization in mice can protect against DSS-induced epithelial damage and inflammation.

### 3.5. Effect of *F. prausnitzii* Colonization on Expression of Colonic Proinflammatory Cytokines in the DSS-Treated Mice

**Figure 6:**
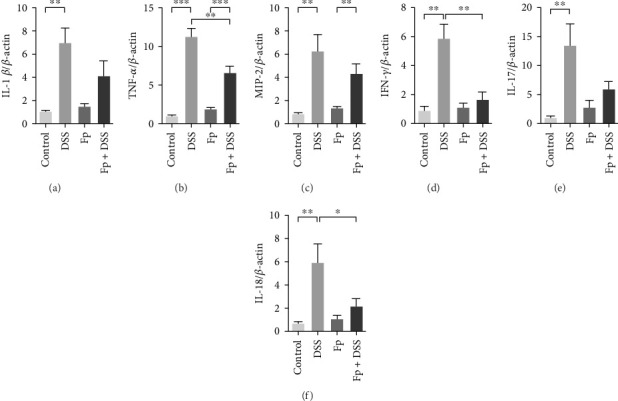
*F. prausnitzii* colonization reduces colonic inflammatory cytokines after DSS induction. (A) IL-1*β*, (B) TNF-*α*, (C) MIP-2, (D) IFN-*γ*, (E) IL-17, and (F) IL-18 (*⁣*^*∗*^*p* < 0.05; *⁣*^*∗∗*^*p* < 0.01; *⁣*^*∗∗∗*^*p* < 0.001).

The expression of TNF-α, IL-1β, IL-17, IL-18, IFN-γ, and MIP-2 normalized to β-actin in the colon tissue is presented in Figure 6. Consisting the histological features representing the increase of inflammatory cells, RNA expression levels of the IL-1β, TNF-α, MIP-2, IFN-γ, IL-17, and IL-18 significantly increased in the DSS group (Figure 6A–F). Compared with the mice in the DSS group, the levels of TNF-α, IFN-γ, and IL-18 significantly decreased in the *F. prausnitzii*-colonized mice after DSS treatment (Fp + DSS group) (Figure 6B,D,F).

### 3.6. *F. prausnitzii* Colonization Alters Mice Gut Microbiota, and DSS Treatment Diminishes the Abundance of *F. prausnitzii*

**Figure 7:**
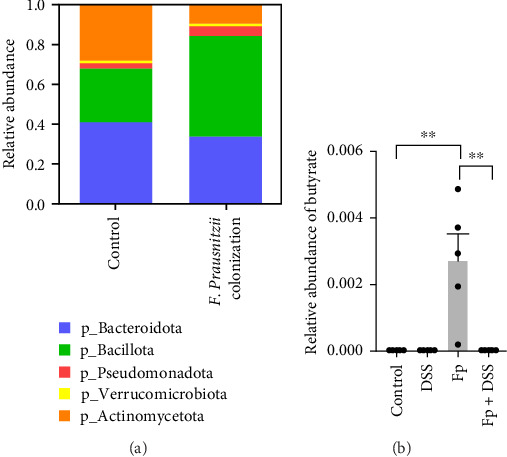
*F. prausnitzii* colonization altered the gut microbiota (A). DSS-induced colitis diminish *F. prausnitzii* abundance in *F. prausnitzii*-colonized offspring mice (B). (*⁣*^*∗*^*p* < 0.05; *⁣*^*∗∗*^*p* < 0.01).

Figure 7 showed the gut microbiota alteration in mice of *F. prausnitzii* colonization and the relative abundance of *F. prausnitzii* in experimental mice prior (day 14) and after (day 17) DSS treatment. The results showed successful colonization of *F. prausnitzii* in the gut of experimental mice with a significant increase of *F. prausnitzii* abundance in *F. prausnitzii*-colonized mice (Fp). Moreover, *F. prausnitzii* colonization altered the gut microbiota to Bacillota dominant and reduced the Bacteroidota and Actinomycedota (Figure 7A). In both *F. prausnitzii*-colonized and wild mice, the DSS treatment significantly reduced the bacterial abundance (Figure 7B).

## 4. Discussion

This presentation successfully establishes a *F. prausnitzii*-colonized mice model and identifies that maternal *F. prausnitzii* colonization promises to colonize *F. prausnitzii* at the offspring's gut. Moreover, the success of *F. prausnitzii* colonization in the gut ameliorates the epithelial damage and inflammation in chemical-induced colitis mice. To our knowledge, this is the first report to show the mice model creating the *F. prausnitzii* colonization and proving the protection effect in DSS-induced colitis. Previous studies have shown that the disruption of gut microbiota–host interactions plays an important role in the pathogenesis of IBD [16]. Among the individual strains of the gut microbiota which affect the IBD development and severity, *F. prausnitzii* is a focus of interest since emerging evidence have shown its potential being the next-generation probiotics [17].

*F. prausnitzii* is a commensal bacterium known to have anti-inflammatory properties, partly through secreting metabolites including butyrate and salicylic acid being able to inhibit the NF-kB signaling pathway and IL-8 production [18]. Previous studies reported that patients with IBD had decreased abundance of *F. prausnitzii* comparing to the healthy people, and the certain bacterial abundance was associated with IBD disease activity [8, 19]. By enhancing the intestinal barrier function and regulating the inflammatory process, administration of *F. prausnitzii* and its supernatant can attenuate the severity of chemical-induced colitis in mice [20–22]. These results implicate that supplement with *F. prausnitzii* or its effective components promising to improve the colitis.

In the present study, we successfully created a *F. prausnitzii*-colonized mice model via maternal *F. prausnitzii* colonization and a 3-day rectal enema. In addition, our findings disclosed that *F. prausnitzii* colonization protects DSS-induced colitis in the novel mice model. To evaluate the severity of colitis, we observed the BWC, stool appearance, colon length, cecum weight, and SAA according to the previous studies [23, 24]. The SAA, as a marker of systemic inflammation, increased in patients with IBD as compared to healthy people [25–27]. In the present study, we confirm again the SAA level induced by DSS was significantly reduced by *F. prausnitzii* colonization in mice. Our results revealed that colonization of *F. prausnitzii* prior to colitis induction significantly minimized the body weight loss, stool consistency, anal bleeding (represented the disease activity index) [28] and systemic inflammation (SAA) and also preserved the colon length and cecum weight.

The improvement of severity of IBD was further reflected by histological examination. Epithelial damage, submucosa edema, and inflammatory cell infiltration are all features that were ascribed to DSS-induced colitis [29]. These changes were all minimized in *F. prausnitzii*-colonized DSS mice in the present study. Goblet cell numbers were also preserved in our *F. prausnitzii*-colonized mice model. Previous studies have shown that there was a reduction of goblet cell numbers and size in mice and human with IBD [30, 31]. It is well-known that goblet cells can produce mucin and strengthen the epithelial barrier [32]. The above histologic findings indicated that the mucosal damage and mucus barrier disruption during colitis could be protected by *F. prausnitzii* colonization.

Moreover, our DSS-induced colitis mice increased the levels of proinflammatory cytokines including IL-1β, TNF-α, IFN-γ, IL-17, and IL-18, which have been shown to increase in IBD patients [33, 34]. As a potent neutrophil chemoattractant and a functional homolog of human IL-8 [35], MIP-2 expression in colonic tissue increased in mice treated with DSS in this study. *F. prausnitzii* colonization could slightly decrease the MIP-2 level. *F. prausnitzii* colonization significantly decreased TNF-α, IFN-γ, and IL-18 in this study. The IL-18 is a Th1 cytokine which can induce IFN-γ and has been detected in the intestinal lesions of active CD patients [36, 37]. Our finding strengthens the reports of previous studies improving the severity of DSS-induced colitis mice models by inhibiting the IL-18 activity [38, 39]. Our results implied that IL-18-mediated signaling may act one of the key components of the cytokine networks influenced by *F. prausnitzii*. Taken together, the present results exhibited the anti-inflammatory property of *F. prausnitzii* for protecting tissue damage during colitis.

Recently, gut microbiota dysbiosis, especially a decrease in the abundance and diversity of specific genera, has been suggested as a pathogenesis for IBD. A decrease of the abundance of *F. prausnitzii* in the gut is associated with the initiation and recurrence of IBDs [8, 18, 40]. Based on the property of short-chain fatty acid production and anti-inflammation effect of *F. prausnitzii*, administration of *F. prausnitzii* or its supernatant promotes to improve the colitis in several mice model studies [10, 20–22, 40]. This presentation first demonstrated that gut colonization with *F. prausnitzii* was sufficient to alter the abundance of gut genera, by which we believe the existence of *F. prausnitzii* in the gut can protect the DSS-induced colitis.

There are some limitations in this study. First, we do not identify the therapeutic effect of *F. prausnitzii* in mice although several previous studies have shown the anti-inflammatory effect of *F. prausnitzii* administration in vitro and in animal study. Second, we do not evaluate other corresponding cytokines such as IL-12/IL-23 which is also related to IBD [41]. Third, we studied the anti-inflammatory effects of *F. prausnitzii* colonization only in the RNA level (qPCR) but not in protein level (such as ELISA method). The complex cytokine networks in the pathophysiology of IBD desire to comprehensive study in the future.

Koch's postulates have been widely taken up across microbiology for the study of pathogenic diseases, including four criteria for identifying the correct pathogen: (i) the bacteria must be present in abundance in every case of the disease and must not be present in a healthy organism; (ii) the bacteria need to be extracted from the host and grown in pure culture and identified; (iii) the bacteria are inoculated back into a healthy host and must then cause the onset of the disease; and (iv) the bacteria must be extracted from the inoculated host and grown in pure culture to be identified as the original causative agent [42]. Because this study did not identify a pathogen of a disease, therefore, we use converse proof by colonizing *F. prausnitzii* into conventional *F. prausnitzii*-negative C57BL/6 mice. This step proves that the *F. prausnitzii* colonization reduces DSS-induced colitis in clinical symptoms, mucosal damage, and cytokine production. The next step is to confirm the *F. prausnitzii* adoptive transfer has the protective effects using the offspring mice as shown in the study results.

In conclusion, the present study has successfully established a *F. prausnitzii*-colonized mice model representing human IBD and convinced that *F. prausnitzii* colonization in mice was sufficient to protect against DSS-induced colitis.

## Data Availability

All data used to support the findings of this study are included within the article, which are available from the corresponding author upon request.

## Supporting Information

Supporting Information Table S1. The primers and references for detecting fecal microbiomes and cytokines.
