# Supplementary material for: Faecalibacterium prausnitzii Colonization Attenuates Gut Inflammation and Epithelial Damage in a DSS-Induced Colitis Mice Model
Source: Mediators Inflamm. 2025 Mar 10;2025:7280675. doi: 10.1155/mi/7280675 (PMC11986197; doi:10.1155/mi/7280675)
Supplement: Supporting Information — Table S1. The primers and references for detecting fecal microbiomes and cytokines. [file 7280675.f1.docx]

**TABLE S1.** The primers and references for detecting fecal microbiomes and cytokines.

| Gene | Primer sequence | Reference |
| --- | --- | --- |
| But_Fp | Forward: AACTTYATYTCCATCAACAAYGC  Reverse: CAGATRAAGCTCTTGCCGC | Designed by our lab. |
| But_2 | Forward: GACCAGCCGTACCAGTTTGATT  Reverse: GCGTTTTGCATTCTGAGTRATG | Designed by our lab. |
| p_Bacteroidota | Forward: GTTTAATTCGATGATACGCGAG  Reverse: TTAASCCGACACCTCACGG | Appl Environ Microbiol. 2015;81:6749–56. |
| p_Bacillota | Forward: GGAGYATGTGGTTTAATTCGAAGCA  Reverse: AGCTGACGACAACCATGCAC | Appl Environ Microbiol. 2015;81:6749–56. |
| p_Actinomycetota | Forward: TGTAGCGGTGGAATGCGC  Reverse: AATTAAGCCACATGCTCCGCT | Appl Environ Microbiol. 2015;81:6749–56. |
| p_Pseudomonadota | Forward: TCAKGTCAGTATGGCCCTTAT  Reverse: CAGTTTTYAGGATTTCCTCCGCC | Appl Environ Microbiol. 2015;81:6749–56. |
| p_Verrucomicrobiota | Forward: GCTAACGCATTAAGTRYCCCG  Reverse: GCCATGCRGCACCTGTCT | Appl Environ Microbiol. 2015;81:6749–56. |
| IL-1β | Forward: GCAACTGTTCCTGAACTCAACT  Reverse: ATCTTTTGGGGTCCGTCAACT | Cell Death Dis. 2018;9:24. |
| TNF-α | Forward: CATCTTCTCAAAATTCGAGTGACAA  Reverse: TGGGAGTAGACAAGGTACAACCC | Acta Pharmacol Sin. 2013;34:1075–83. |
| MIP-2 | Forward: TGTCATGCCTGAAGACCTGCC  Reverse: AACTTTTTGACCGCCCTTGAGAGTGG | Infect Immun. 2012;80:2076–88. |
| IFN-γ | Forward: GCCATCAGCAACAACATAAGCGTC  Reverse: CCACTCGGATGAGCTCATTGAATG | EMBO Rep. 2017;18:39–47. |
| IL-18 | Forward: CTGAAGAAAATGGAGACCTGGAA  Reverse: TTCACAGAGAGGGTCACAGCC | Biomed Pharmacother. 2018;102:670-80.  J Immunol. 2006;176:949–56. |
